# Conversion of a Surgical Elbow Arthrodesis to Total Elbow Arthroplasty

**DOI:** 10.1155/2015/578189

**Published:** 2015-02-26

**Authors:** Dominik Rog, Lee M. Zuckerman, Barth Riedel

**Affiliations:** Department of Orthopaedic Surgery, Loma Linda University Medical Center, 11406 Loma Linda Drive, Suite 218, Loma Linda, CA 92354, USA

## Abstract

Arthrodesis of the elbow joint addresses pain due to intra-articular pathology, but with significant functional limitations. Loss of motion at the elbow is not completely compensated by the wrist and shoulder joints and elbow fusion is thus purely a salvage procedure. Advances in joint arthroplasty have allowed surgeons to address the functional limitations of arthrodesis, but despite these advances the elbow is still one of the joint replacements with higher complication rate. Conversion of a joint fusion to arthroplasty has been reported for the hip, knee, shoulder, and ankle. The takedown of a surgically fused elbow was reported in German literature in 2013. We present the first such case report in the English literature with a 49-year-old male whose status is elbow fusion performed for trauma 31 years prior.

## 1. Introduction

The tradeoff for pain relief and stability provided by surgical arthrodesis is complete loss of motion at the joint. In certain joints, arthroplasty may provide equivalent pain relief while addressing the decrease in functional capacity. Recent advances in joint replacement, such as anatomic designs and improved polyethylene, have led to an interest in the conversion of surgically fused joints. Takedown of hip and knee fusions with conversion to arthroplasty has been well described in the orthopedic literature [1–6]. Conversion of painful glenohumeral fusion to total shoulder arthroplasty has been reported as early as 1975 [7–9]. More recently, total ankle arthroplasty has been reported as a viable treatment for a failed ankle fusion [10, 11].

Fusion of the elbow joint is an option for a relatively young patient with unilateral posttraumatic osteoarthritis who requires a stable joint. This, however, must be considered a salvage option as the wrist and shoulder joints are unable to completely compensate for motion loss at the elbow [12]. Fusion of the elbow joint, furthermore, is not always completely effective in eliminating pain [13]. Despite the profound functional limitations of an elbow arthrodesis, there has only been one published report of takedown of an elbow arthrodesis to arthroplasty by Burkhart et al. in Germany [14]. We present the first case in the English literature of a conversion from an elbow arthrodesis to a total elbow arthroplasty.

## 2. Case Report

**Figure 1:**
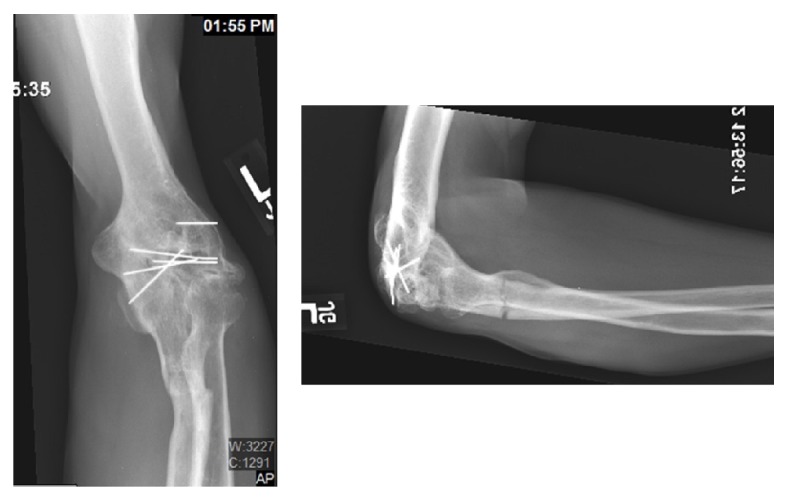
Plain radiographs of the elbow at the time of presentation revealed an elbow arthrodesis at 90 degrees with retained hardware and a minimally displaced transverse proximal ulnar shaft fracture.

A 49-year-old male presented with a complaint of pain in the left proximal forearm after a fall. The patient had a history of left elbow arthrodesis performed for posttraumatic arthritis at the age of 18. On physical examination he was tender at the proximal ulna. He had no active flexion or extension at his elbow, which was fused at 90 degrees but achieved 40 degrees of pronation and 60 degrees of supination. His motor and sensory exam was normal at the hand. Radiographs of the forearm and the elbow revealed an elbow arthrodesis at 90 degrees with retained hardware and a minimally displaced proximal ulnar shaft fracture (Figure 1). A decision was made to treat his ulnar shaft fracture closed in a cast, and he subsequently developed a hypertrophic nonunion. At his clinic visit three months after the fall, surgical options for the ulna nonunion were discussed with the patient. We proceeded with conservative treatment for an additional three months, with worsening motion through the nonunion site. He revealed that he was unhappy with the functional limitations of his elbow arthrodesis and inquired about the possibility of converting it to an arthroplasty. The risks of elbow arthroplasty were discussed with the patient at length. Increasing the functional capacity of his arm was his ultimate goal, and understanding that he faced a likely operation for the ulna nonunion, the patient wished to proceed. Due to the patient's prior surgery and history of trauma, as well as risk of infection, we chose to avoid multiple surgeries and combine the repair of nonunion and the conversion of elbow arthrodesis to arthroplasty into one procedure. The stem of the ulnar component would thus act as an intramedullary device.

**Figure 2:**
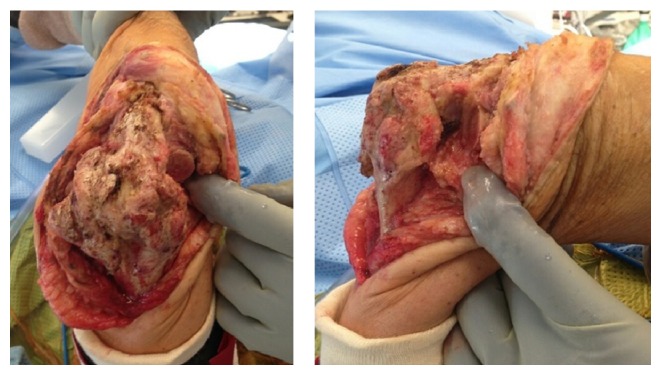
Intraoperative photographs of the fusion mass after radial head resection, prior to wedge osteotomy.

**Figure 3:**
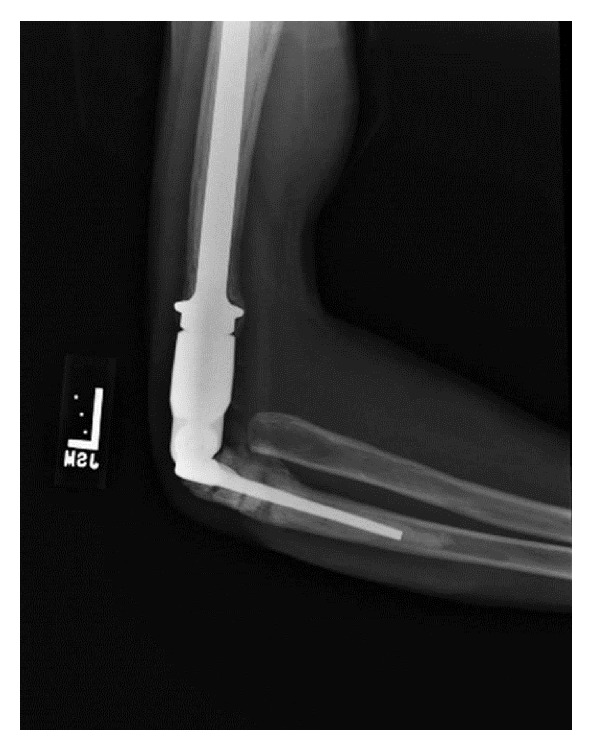
Plain radiograph taken at the patient's 4.5-month appointment reveals a healed ulna nonunion and a stable total elbow prosthesis without signs of loosening.

In the operating room the patient was placed in a supine position and a posterior incision centered over the elbow was performed. A prior muscle flap that was used for soft tissue coverage at his index procedure had to be elevated. The ulnar nerve was encased in scar tissue and required a meticulous neuroplasty. A triceps splitting approach to the elbow joint was then performed and multiple buried pins were removed from the humerus [15]. A wedge osteotomy of the arthrodesis site was then performed and the fusion taken down (Figure 2). This was performed at the apex of the arthrodesis site with the humeral cut at 90 degrees to the long axis of the humerus and the ulnar cut at 45 degrees to the long axis of the ulna. The cuts were done in this manner to better accommodate the stems of the prosthesis. Resection of the humerus was greater than normal to allow for appropriate range of motion (ROM) of the elbow without undue tension on the neurovascular structures, which had been in this position for over 30 years. Resection of the radial head was performed as it was markedly arthritic. After preparation of the canals, a Stryker distal humeral replacement system was used to perform the total elbow arthroplasty (MRS (Stryker, Kalamazoo, MI)). Intraoperatively, the patient had full flexion and extension of the elbow and full pronation and supination. His muscles were properly tensioned without undue strain on the neurovascular structures. The patient's ulnar nonunion was also addressed with bone graft taken from the resected radial head. He had an uncomplicated hospital course and was allowed full ROM on postoperative day #2. At his 4.5-month appointment, the patient was achieving 0–110° elbow active elbow flexion/extension, as well as nearly full forearm rotation. He was experiencing minimal pain and was happy with the function of his prosthesis. The patient was able to return to work with an elbow brace that he locked at work. Radiographs showed a healed ulna nonunion and a stable total elbow prosthesis without signs of loosening (Figure 3). Multiple attempts to contact the patient for further follow-up have been unsuccessful.

## 3. Discussion

Posttraumatic elbow arthritis in the young and active population is a challenge to treat. The main goal of surgical options such as arthroscopic or open debridement with capsular release, interposition arthroplasty, or partial joint replacement is to restore pain-free range of motion and provide a functional joint [16]. End-stage osteoarthritis can be managed with total elbow arthroplasty or arthrodesis. Most activities of daily living can be performed with extension limited at 75° and flexion at 120° [17]. Fusion of most joints is well compensated by adjacent articulations. The shoulder and wrist, however, cannot fully compensate for immobilization of the elbow at any angle to allow all activities to be completed [12].

Total elbow arthroplasty provides excellent range of motion, but with a lifetime weight limit. The tradeoff, therefore, is between a strong and stable joint with significant functional limitations and increased function with activity limitations. A patient who was provided a stable joint through arthrodesis when he was young may desire an increase in the functional capacity of his upper extremity if his occupational demands were to change. Experience with conversion of fusions at the hip and knee has shown that a satisfactory result is possible for patients who desire to regain motion of their surgically fused joints. The treatment of spontaneous ankylosis of the elbow with total elbow arthroplasty suggests that this is feasible in the surgically fused elbow [18–20]. In a recent German case report, Burkhart et al. describe a conversion in a 44-year-old patient who had been fused for 7 years.

The case presented in this report is the first documented conversion of elbow arthrodesis to total elbow arthroplasty in the English literature and highlights several unique challenges. Our patient's elbow was fused 31 years ago secondary to trauma. Chief among concerns during preoperative planning after such a prolonged arthrodesis was preventing tension on neurovascular structures. This was addressed by planning a wedge resection osteotomy, which then necessitated a specialized implant to accurately tension the elbow. A muscle flap was used for soft tissue coverage during his initial trauma, which considerably increased the difficulty of the dissection. Similar to the importance of abductor function in the outcome of hip ankylosis conversion, the integrity of muscles controlling elbow function needs to be considered. Preoperatively, the patient had maintained functional pronosupination, and when he was asked to fire his tricep, a robust contraction of the muscle belly could be felt. Through therapy, he achieved active flexion and extension of the elbow soon after the operation.

Conversion of elbow arthrodesis to total elbow arthroplasty is feasible in carefully selected patients who are unsatisfied with the functional limitations of a fusion. The duration of fusion and any alterations in anatomy from prior surgeries must be taken into account during preoperative planning.
